# Supplementary material for: A high-resolution single-molecule sequencing-based Arabidopsis transcriptome using novel methods of Iso-seq analysis
Source: Genome Biol. 2022 Jul 7;23:149. doi: 10.1186/s13059-022-02711-0 (PMC9264592; doi:10.1186/s13059-022-02711-0)
Supplement: Supplementary file 4 — Additional file 4: Review history. [file 13059_2022_2711_MOESM4_ESM.docx]

Review History

**First round of review**

**Reviewer 1**

**Are you able to assess all statistics in the manuscript, including the appropriateness of statistical tests used?** Yes, and I have assessed the statistics in my report.

**Comments to author:**

The authors present an enriched transcriptome annotation for Arabidopsis thaliana, AtTRD3, based on IsoSeq data.
The study is well designed as it includes a variety of tissues and conditions. The analysis seems rigorous, applicable to other studies, and the text is clear. Figures are not as clear. The manuscript would be improved by giving more context about the goal to reach and how the new transcriptome compares to that goal. Biological systems being noisy, a complete list of every single transcript, mature and immature, that can ever be produced by an Arabidopsis cell (including mutants) may not actually be that informative.

Major comments:
1. I am unsure about the wisdom of including RNA processing mutants. There is a risk of introducing lots of "non-physiological" transcripts (transcription intermediates, aberrant transcripts) that would add noise to the annotation and potentially affect the accuracy of short-read isoform quantification with Salmon/Kallisto. It would help if the authors included some metrics about how many new transcripts are contributed by a particular condition/tissue. If most new transcripts have little support from physiological conditions, maybe they should not be included. If a physiological condition contributes lots of new transcripts, it could be a sign that more attention should be paid to it/it should be sequenced more deeply. The fact that 40% of transcripts are non-productive suggests a lot of the new annotation may be transcriptional noise.
2. The sequencing depth is very shallow (13M reads total) which biases the analysis towards highly expressed transcripts. It is still a lot of sequencing for Pacbio before the 8M SMART cells were introduced, and I am not requesting additional sequencing. However a saturation curve analysis would be very useful to gauge the "completeness" of the new annotation. Are we 90% there? 10% there? What would be the ideal sequencing depth if money/technology was no object? This can be discussed in light of current PacBio and Nanopore throughput.
3. Related to the point above, there needs to be some discussion of what a "complete" annotation should look like. The criteria for including new transcripts in this study are very relaxed (1 good quality supporting read), and susceptible to "transcription noise" (in particular with the inclusion of RNA processing mutants). A natural suggestion would be to consider relative abundance: isoforms that make up <1% of a gene's transcripts in all conditions may not be relevant, and could just be noise? 
4. To help judge the importance of the new transcript annotation, it would help to quantify how much more expression is now accounted for. For example, did existing isoforms account for 90% of expression (based on short or long-read counts), and now we can capture 99%?
5. Fig2 is very difficult to interpret. Making the Venn diagram in Fig2A (and S3) area-proportional would help a lot in visualising the magnitude of the overlaps. Panel labels in the legend do not always match those of the figure. The y-scale in H should start at 0, it's very misleading otherwise. "Number of errors" in C-F isn't very informative because it doesn't allow to compare C to E and D to F. Could these be expressed as percentages, and the same y-scale kept across plots?
6. I am also not sure about the meaning of the y-scale on Fig3, "instances". Is this appropriately scaled so we can compare the height of the various lines? A label of the lines on the plot would also be very helpful, rather than having to read the whole legend and memorise the colour-name scheme.
7. Fig4: 3D pie charts are generally advised against, as it makes it difficult to fairly compare categories. 
8. Fig5 should definitely plot all the data. Outliers can have large impacts on correlation. 
9. The validation using the cold- and light-induced data set does not seem complete: a handful of cherry-picked examples are shown to illustrate differential isoform accumulation, but we don't know how many of the 2251 testable TSS genes switch isoforms, or what is the overlap with the 82 genes that were previously shown to switch. A Differential Transcript Usage analysis would be much more informative than a few isolated illustrations.

**Reviewer 2**

**Are you able to assess all statistics in the manuscript, including the appropriateness of statistical tests used?** Yes, and I have assessed the statistics in my report.

**Comments to author:**

This manuscript describes the assembly, guided by extensive PacBio sequencing of full-length Arabidopsis cDNAs, of an updated collection of Arabidopsis transcripts. The PacBio sequence collection was derived from a range of tissues and physiological states, and included samples from a selected few mutants. Most transcript variants that arise via alternate promoter, splice site, and poly(A) site usage should be represented in these samples. The authors use the PacBio collection along with extant annotations to derive the final "new" transcriptome database.

The concept behind this study - to link different possible transcript variants that may be discovered by short-read sequencing into coherent, connected whole transcripts - is important, and AtRTD3 should be a resource that may be used by the community to test more discerning hypotheses regarding gene regulation in Arabidopsis. The authors go to great lengths to minimize the errors that often are seen in transcriptomics databases. Chief among these are the inclusion of sequencing-derived splice site mistakes; the strategies used to filter these out of the final database are appropriate and the execution of the filtering conducted in a logical manner. Importantly, the collection of PacBio sequences is large enough to permit a sizeable (albeit not comprehensive) probabilistic approach to defining transcript ends.

That being said, this manuscript is designated as a Research contribution (as opposed to "new methods or software"). While the upgrading of the transcriptomics resource could be an important contribution for the Arabidopsis community, there is not much new presented in this study about aspects of gene expression in Arabidopsis, nor of specific aspects of gene expression or regulation. To be sure, there is a potential for considerable hypothesis testing. There is a good sampling of different tissues and of different physiological conditions, but these are sources for RNA, or are selectively drawn upon to validate aspects of the AtRTD3 database.

In addition to this general comment, the following specific remarks should be considered:

1. On pp. 19-20, many transcripts are suggested to be nonsense-mediated decay (NMD) substrates, based on sequence characteristics often seen in NMD substrates (described in detail in reference 53). Included in the samples assembled for library construction are those prepared from mutants altered in RNA quality control (upf1, upf3, xrn2, xrn3, and xrn4). The authors should use data from these mutants to support the computational assignments, or at least augment them (for example, classify potential non-productive transcripts according to whether they are seen more prominently in the RNA quality-control mutants).

2. While some attention is paid to possible NMD substrates, nonstop RNAs are not mentioned or identified. These would be apparent as transcripts with TES within protein-coding regions. This is an important subset of non-productive transcripts and should be identified and annotated in AtRTD3.

3. In the methods, it is stated that one sample was prepared using RNA from isolated nuclei. While I can appreciate that this sample would expand the database to include unspliced or partially-spliced transcripts, it is not clear how this will inform a database of transcripts to be used by the Arabidopsis community. Intronic transcript sequences arising from nuclear RNA may or may not be relevant to aspects of gene regulation (alternative splicing and the like), and it seems as if this will only bloat the complete database. Perhaps transcript isoforms present largely or exclusively in the nuclear RNA samples can be so annotated, and perhaps segregated into a separate database.

4. There have been other reports describing chimeric transcripts in Arabidopsis (Duc. et al., PLoS Genet. 2013 Oct;9(10):e1003867 and Pontier et al., Life Sci Alliance. 2019 May 29;2(3):e201900393 come to mind). These reports might used to validate some of the chimeric RNAs reported in AtRTD3.

5. TES's were validated using signals/motifs derived from characterizations of mRNA polyadenylation in mammals (pp. 16-17, Fig. 3E and F, noted in Table S6). Since plant 3' processing signals are somewhat different, it would be better to validate using different motifs or approaches.

6. Several "new" transcripts are presented in the AtRDT3 database. Some additional study of these would seem to be warranted. For example, can possible function for predicted proteins be assigned? Is there evolutionary conservation of novel proteins or non-coding RNAs? Is there evidence, in other genome-scale or proteomic studies, of translation of these novel transcripts? Are there hints of tissue-specific or other regulated aspects of the expression of these transcripts?

**Authors Response**

**Point-by-point responses to the reviewers’ comments:**

Response to Reviewers’ comments

Our responses to the Editor and Reviewers are in bold italics and the changes made to the manuscript are in red.

***A) We do not agree that many of the comments of the referees are serious concerns because many reflect a misunderstanding of what the reference transcript dataset is and how it should be used to enable accurate differential gene expression and differential alternative splicing analysis of RNA-seq experiments. As a reference to be used to analyse RNA-seq data, it has to be as comprehensive as possible and the constituent transcripts must be as accurate as possible. The first key step in downstream analysis is accurate transcript quantification by Salmon or kallisto, both of which require a reference transcriptome or RTD. We identified factors which negatively affect accurate transcript quantification (redundancy, transcript fragments, missing transcripts, mis-assembled transcripts etc) (Zhang et al., 2017). Many other studies from highly respected groups in the field of RNA-seq data analysis have obtained similar results (Soneson et al, 2019; Hsieh et al, 2019; Ma et al, 2019; Varabyou et al, 2020), which include the groups of Mark Robinson (EdgeR), Carl Kingsford (Salmon), and Steven Salzberg (Bowtie, Tophat, HISAT, Stringtie etc.). The novel methods and approaches in this paper (which both referees see the value of) are all about ensuring that the transcripts are accurate and supported such that AtRTD3 is optimised to contain real transcripts and artefacts are kept to a minimum. As such, it is completely justified to include all transcripts in the reference. The second consideration is how Salmon and Kallisto determine transcript abundance. If a transcript is very rare (e.g. a splicing intermediate where one or more introns have not been spliced out at the time of RNA extraction), it will have low abundance in the cell and this will be reflected in the quantification of the transcript. In essence, it does not matter whether the reference contains transcripts which are likely to be of very low abundance (as long as they are accurate and unique) because the RNA-seq data and associated quantification methods, such as Salmon and Kallisto will resolve this. Our studies so far show that their very low level will have little effect on the accurate quantification of the main isoforms from the gene as illustrated by the improvement of quantification accuracy at the alternative splicing event level using our HR RT-PCR data (Figure 5). More importantly, in specific studies (different treatments, stresses, tissue- or cell-specific experiments), some of these transcripts may have increased abundance; if they are not in the reference, their effects will not be quantified for that RNA-seq experiment. On the other hand, an RTD with selective/minimum transcript diversity will have limitations as a universal reference for Arabidopsis RNA-seq data analysis. It is the combination of the comprehensive RTD and accurate quantification of RNA-seq data that will generate insights and new knowledge. Therefore, the reference should be as comprehensive as possible containing accurate high-quality transcripts which our methods produce. With this basic understanding and philosophy, we believe that many of the points raised by the reviewers can be easily addressed.***

***Soneson et al, 2019 https://www.life-science-alliance.org/content/2/1/e201800175; Hsieh et al, 2019 https://www.nature.com/articles/s41598-019-44499-3; Ma et al, 2019 https://www.sciencedirect.com/science/article/pii/S2405471219303813; Varabyou et al, 2020 https://genome.cshlp.org/content/31/2/301.***

Reviewer reports:

Reviewer #1: The authors present an enriched transcriptome annotation for Arabidopsis thaliana, AtTRD3, based on IsoSeq data.

The study is well designed as it includes a variety of tissues and conditions. The analysis seems rigorous, applicable to other studies, and the text is clear. Figures are not as clear. The manuscript would be improved by giving more context about the goal to reach and how the new transcriptome compares to that goal. Biological systems being noisy, a complete list of every single transcript, mature and immature, that can ever be produced by an Arabidopsis cell (including mutants) may not actually be that informative.

***B) We thought that the goal had been clearly stated – to generate the most comprehensive and accurate reference transcript dataset for Arabidopsis to enhance downstream RNA-seq analysis. We have revised the end of the Introduction section to make this clear (page 7-8). The point about having a complete list of transcripts not being that informative is explained in A) above as a function of a reference for RNA-seq analysis of ever more refined plant material (tissue and cell types, single cells etc).***

Major comments:

1. I am unsure about the wisdom of including RNA processing mutants. There is a risk of introducing lots of "non-physiological" transcripts (transcription intermediates, aberrant transcripts) that would add noise to the annotation and potentially affect the accuracy of short-read isoform quantification with Salmon/Kallisto.

***C) RNA processing plays key roles in the regulation of gene expression and downstream protein expression. Some of the isoforms detected in the mutants may be important in specific conditions such as different abiotic or biotic stresses. Capturing these isoforms will aid identification of RNA processing events underpinning the regulation of stress responses of particular genes. For example, under normal conditions, some of the unproductive alternatively spliced isoforms containing PTCs transcripts are readily detected in wild-type plants and their levels can vary greatly from gene to gene (Kalyna et al., 2012; Raxwal et al., 2020). On the other hand, many transcripts containing PTCs will have low abundance and will not impact quantification of other more abundant isoforms (see A above***).

It would help if the authors included some metrics about how many new transcripts are contributed by a particular condition/tissue.

***D) We have now provided a new Supplementary Table and Figure giving the number of genes and transcripts contributed by each sample (Table S7 and Fig. S5) and described in the text (page 18-19).***

If most new transcripts have little support from physiological conditions, maybe they should not be included. If a physiological condition contributes lots of new transcripts, it could be a sign that more attention should be paid to it/it should be sequenced more deeply.

***E) Our quality control methods are designed to include transcripts in AtRTD3 that are as accurate as possible and supported by reads. The problem is that given the massive range of biological questions being addressed by RNA-seq experiments, it is impossible to know which transcripts may be important at any specific time or condition. Therefore, the strategy has to be to have a reference that is complete and accurate as possible (again bearing in mind that low abundance transcripts are unlikely to affect quantification of main isoforms).***

The fact that 40% of transcripts are non-productive suggests a lot of the new annotation may be transcriptional noise.

***F) We are not surprised by the figure of 40% of transcripts being unproductive. In the previous Arabidopsis RTD (AtRTD2 - Zhang et al., 2017), 26% of the 82k transcripts were unproductive. In AtRTD3 with substantially more transcripts (169.5k), 40% are unproductive which means that AtRTD3 actually has a higher number of protein-coding variants.***

***Secondly, we do not believe that the unproductive transcripts are necessarily transcriptional noise but more a reflection of innate complexity of the transcriptome as a result of transcriptional/post-transcriptional regulation such as alternative splicing, alternative transcription start or polyadenylation sites, which are all active mechanisms that regulate the expression levels of productive coding transcripts. We have applied high stringency of quality control filtering to generate the transcripts that make up the RTD. Unproductive but real transcripts include transcripts where the CDS is affected (e.g. AS events) and reflect regulation of splicing and expression.***

***Finally, Varabyou et al. (2021) described “transcriptional noise” referring to mis-assembled transcripts such as transcripts with incorrect splice junctions etc.. We have employed stringent filters to remove this type of “transcriptional noise”.***

2. The sequencing depth is very shallow (13M reads total) which biases the analysis towards highly expressed transcripts. It is still a lot of sequencing for Pacbio before the 8M SMART cells were introduced, and I am not requesting additional sequencing. However a saturation curve analysis would be very useful to gauge the "completeness" of the new annotation. Are we 90% there? 10% there? What would be the ideal sequencing depth if money/technology was no object? This can be discussed in light of current PacBio and Nanopore throughput.

***G) We have generated a saturation curve of genes and transcripts (new Supplementary Table S8 and Supplementary Fig S6. The number of genes in AtIso increases significantly until 8 samples are added and then increases more slowly. The number of transcripts continues to increase as samples are added and so transcript numbers are not yet saturated (page 18-19).***

3. Related to the point above, there needs to be some discussion of what a "complete" annotation should look like. The criteria for including new transcripts in this study are very relaxed (1 good quality supporting read), and susceptible to "transcription noise" (in particular with the inclusion of RNA processing mutants). A natural suggestion would be to consider relative abundance: isoforms that make up <1% of a gene's transcripts in all conditions may not be relevant, and could just be noise?

***H) Here we would like to clarify that the criteria for inclusion of an Iso-seq transcript of one good supporting read is incorrect. We require at least one Iso-seq read that has no sequencing errors in the region flanking a splice junction to define a supported splice junction. This information can come from multiple reads which in combination define all of the SJs of the transcripts of a gene. Our comparison with the splice junctions obtained using short reads show that by using only one Iso-seq read with no mismatches in the region of the splice junction, we can obtain sufficient reduction of false splice junctions as illustrated by Figure 2E. In addition, 80% of the splice junctions from Iso-seq data have been validated by short reads. In terms of defining transcriptional start and end sites of transcripts, at least two Iso-seq reads were required for genes with low coverages. The number is much higher for genes with higher coverages.***

***Due to the relatively limited coverage of Iso-seq data for some genes (e.g. there are 6.5K genes that only have one read and 9K genes with no more than two reads) increasing the threshold risks removing valid splice junctions, and more importantly would exclude 20% to 30% of the genes with Iso-seq evidence. Thus, we believe that by defining each splice junction on the basis of having at least one read with no errors flanking the SJ strikes a good balance between the coverage and accuracy. As more Iso-seq data becomes available, the same pipeline can be used to explore the increase the thresholds without a significant loss of the gene coverage.***

***The suggestion to use relative abundance to remove all transcripts with, say, <1% of expression of a gene’s transcripts in all conditions is not feasible as 1) we do not cover all conditions, 2) we do not know under which conditions as isoform may be relevant (see A above) and 3) most existing RNA-seq experiments are from mixtures of tissues and cell types and rare transcripts will have low abundance; however, such transcripts may be abundant in certain cell types and single cell transcriptomics is highly likely to benefit from a comprehensive RTD. We also carried out additional analysis using SQUANTI3, which shows that the novel transcripts in AtRTD3 (compared to Araport11) are high quality with overall better support for transcriptional starting sites, which is consistent with our existing assessment (page 19-20).***

4. To help judge the importance of the new transcript annotation, it would help to quantify how much more expression is now accounted for. For example, did existing isoforms account for 90% of expression (based on short or long-read counts), and now we can capture 99%?

***I) We do not think that this is a valid point. The new transcript annotation is not about increasing overall expression detected but resolving the details of expression more accurately in terms of contribution from different transcript isoforms. The simplest example is that if an isoform is missing, its signal (k-mers in Salmon and Kallisto) will be allocated to other isoforms thereby skewing quantification (see Zhang et al., 2017).***

***If we compare Araport11 to AtRTD3, AtRTD3 has four times the number of transcripts which are accurately defined in SJs, TSS and TES and novel SJs defining new AS events. With this increased diversity, we are discovering more about the real level of complexity of the transcriptome that has been missed to date.***

***We can compare read mapping rates with the proviso that judging expression on the basis of % of reads mapped is a very crude measurement which does not reflect accuracy of the transcriptome. We show below the mapping rate from Salmon for a number of RNA-seq libraries generated from leaf material. It shows that AtRTD3 has a mapping rate increase of around 1% from 97.59% to 98.46% compared to Arapport11. The increased read mapping reflects the increased definition of transcripts and differentiating reads mapping to novel splice junctions, intron retention events, and defined ends. Importantly, although Araport11 has a 97.5% read mapping rate, it still significantly lacks transcript diversity and misses 46K (25%) splice junctions in AtRTD3.***

***sample Araport11 atRTD3***

***792_LIB5958_LDI4774_TGACCA_L003 97.46% 98.47%***

***792_LIB5984_LDI4800_GCCAAT_L006 97.52% 98.27%***

***801_LIB6010_LDI4825_TGACCA_L001 97.66% 98.44%***

***801_LIB6010_LDI4825_TGACCA_L002 97.63% 98.40%***

***801_LIB6010_LDI4825_TGACCA_L003 97.64% 98.42%***

***805_LIB5958_LDI4774_TGACCA_L003 97.61% 98.69%***

***805_LIB5984_LDI4800_GCCAAT_L006 97.71% 98.50%***

***R1792_LIB5958_LDI4774_TGACCA_L003 97.48% 98.57%***

***R1792_LIB5984_LDI4800_GCCAAT_L006 97.56% 98.37%***

***Although the 1% increase of mapping rate appears modest, it could contribute significantly to the identification of expression of relatively lowly expressed genes and improved quantification at transcript isoform levels. Also, genes and transcripts expressed in leaf are well studied and annotated and the improvement is likely to be more significant for less studied tissues/conditions.***

5. Fig 2 is very difficult to interpret. Making the Venn diagram in Fig2A (and S3) area-proportional would help a lot in visualising the magnitude of the overlaps.

***J) Venn diagrams in Fig. 2A and S3 are now area proportional.***

Panel labels in the legend do not always match those of the figure. The y-scale in H should start at 0, it's very misleading otherwise. "Number of errors" in C-F isn't very informative because it doesn't allow to compare C to E and D to F. Could these be expressed as percentages, and the same y-scale kept across plots?

***K) We apologise for the mix-up in labelling in the Figure legend and this has been corrected. We are grateful for the reviewer’s suggestion to plot the two classes of SJs together (see Fig. 2C-E). This simplifies the figure and clearly shows the massive difference between the high and low quality SJs and that without quality control Iso-seq will generate many false SJs which will carry through to transcripts.***

6. I am also not sure about the meaning of the y-scale on Fig3, "instances". Is this appropriately scaled so we can compare the height of the various lines? A label of the lines on the plot would also be very helpful, rather than having to read the whole legend and memorise the colour-name scheme.

***L) We have changed “instances” to “No. of motifs identified” and include a label for the lines.***

7. Fig4: 3D pie charts are generally advised against, as it makes it difficult to fairly compare categories.

***M) We do not think that there is a need to change the Figure. Although 3D, each segment is labelled with the number and % of each type of transcript.***

8. Fig5 should definitely plot all the data. Outliers can have large impacts on correlation.

***N) We now provide a new Supplementary Figure S14 which plots the splicing ratios and includes the outliers; we have kept the original Figure as Figure 5 and refer to both in the text.***

***The plots in Figure 5 illustrate the reduced spread of consistency between the experimentally defined splicing ratio and that calculated from RNA-seq data using different references. Including the small number of outliers reduces the plots currently seen to a much smaller area making it more difficult to see the tightening of consistency. While the plots are visually illustrative, the correlation coefficients show the improvement. In addition, Spearman correlations are unaffected by outliers. We clearly state in the legend that the outliers are included in the correlation coefficient calculations.***

9. The validation using the cold- and light-induced data set does not seem complete: a handful of cherry-picked examples are shown to illustrate differential isoform accumulation, but we don't know how many of the 2251 testable TSS genes switch isoforms, or what is the overlap with the 82 genes that were previously shown to switch. A Differential Transcript Usage analysis would be much more informative than a few isolated illustrations.

***O) We understand this comment; we had tried to keep the description brief of the analysis by which we arrived at the examples of Differential TSS and TES usage. We have now changed the text to give a more complete description in the manuscript (and see below).***

***Answers to the specific points: the 2251“testable” genes have alternative TSS. 82 of these overlap with 220 genes shown to have blue light induced differential TSS usage showing good agreement even though blue light treatment was not included in our samples. In the re-analysis of the cold time-series data 583 of these genes had cold-induced isoform switches which were further analysed to identify TSS usage (see below). It is likely that others will also switch under different conditions and AtRTD3 can aid their identification.***

***To identify differential TSS and TES usage in the cold we re-analysed our cold response time-series (26 time-points; Calixto et al., 2018) using AtRTD3. We focussed on the isoform switch data but have the differential gene/transcript, differential alternative splicing and DTU analyses. However, the DTU analysis does not identify isoform switches; for that we needed TSIS. However, the isoform switch analysis does not distinguish whether an isoform switch is due to differential TSS and/or TES usage or to AS in the transcripts. To identify the TSS/TES usage, we took the genes with significant isoform switches in genes with more than one TSS or TES which were >100 bp apart. We then identified those genes with the largest differences in TPM between isoforms and with high negative correlation values and analysed individual isoform pairs to select only those with no AS. By only taking examples of TSS/TES switches with no AS, we arrived at a selection of examples to illustrate how AtRTD3 can be used to define differential TSS and TES.***

***It is important to point out that many groups still do not analyse RNA-seq data at the transcript level but only at the gene level, do not routinely include differential AS analysis, do not have time-series data with which to identify isoform switches and continue to use TAIR10 or Araport as references which do not have variant TSS and TES data (quite apart from their other limitations). Therefore, we have clearly illustrated how AtRTD3 can be used to analyse differential TSS and TES usage and by supplying AtRTD3, other groups can now re-analyse existing or plan new RNA-seq experimental data.***

Reviewer #2: This manuscript describes the assembly, guided by extensive PacBio sequencing of full-length Arabidopsis cDNAs, of an updated collection of Arabidopsis transcripts. The PacBio sequence collection was derived from a range of tissues and physiological states, and included samples from a selected few mutants. Most transcript variants that arise via alternate promoter, splice site, and poly(A) site usage should be represented in these samples. The authors use the PacBio collection along with extant annotations to derive the final "new" transcriptome database.

The concept behind this study - to link different possible transcript variants that may be discovered by short-read sequencing into coherent, connected whole transcripts - is important, and AtRTD3 should be a resource that may be used by the community to test more discerning hypotheses regarding gene regulation in Arabidopsis. The authors go to great lengths to minimize the errors that often are seen in transcriptomics databases. Chief among these are the inclusion of sequencing-derived splice site mistakes; the strategies used to filter these out of the final database are appropriate and the execution of the filtering conducted in a logical manner. Importantly, the collection of PacBio sequences is large enough to permit a sizeable (albeit not comprehensive) probabilistic approach to defining transcript ends.

These introductory remarks are very good and supportive comments which capture the essence of what generating the reference is about.

That being said, this manuscript is designated as a Research contribution (as opposed to "new methods or software"). While the upgrading of the transcriptomics resource could be an important contribution for the Arabidopsis community, there is not much new presented in this study about aspects of gene expression in Arabidopsis, nor of specific aspects of gene expression or regulation. To be sure, there is a potential for considerable hypothesis testing. There is a good sampling of different tissues and of different physiological conditions, but these are sources for RNA, or are selectively drawn upon to validate aspects of the AtRTD3 database.

***P) We submitted this manuscript to the “Long-read Sequencing” special issue, where “Genome Biology will highlight the advances in applications and methods of long-read sequencing in related areas”. We believe that our work represents a significant and innovative advance of long-read sequencing methods and meets the brief of the special issue. In addition, to the development of new computational methods for data analysis which is a significant “research” contribution the paper contains biological innovations in the complexity and diversity of transcripts and that examination of differential TSS and TES is now possible with AtRTD3.***

***In general, the new reference transcriptome has massive potential to impact plant biology and demonstrate the power of routinely working at the transcript level. The new RTD is far more accurate and comprehensive that the (still) widely used TAIR10 and Araport11 transcriptomes and when used by the community will greatly enhance the quality of differential gene/transcript and AS analyses. The methods that we have developed are also not restricted to Arabidopsis or plants.***

***The reviewer suggests that the wide range of samples could be exploited. The problem with this is that for the most part the samples are mixtures of RNA from related samples (e.g. different exposure times, developmental stages etc) and there is only one biological repeat of each sample and so are not quantitative. Again, the strategy is to capture diversity accuracy in the transcript set in the RTD (see section A above) which is then used to analyse replicated RNA-seq experiments to address specific physiological and developmental questions.***

In addition to this general comment, the following specific remarks should be considered:

1. On pp. 19-20, many transcripts are suggested to be nonsense-mediated decay (NMD) substrates, based on sequence characteristics often seen in NMD substrates (described in detail in reference 53). Included in the samples assembled for library construction are those prepared from mutants altered in RNA quality control (upf1, upf3, xrn2, xrn3, and xrn4). The authors should use data from these mutants to support the computational assignments, or at least augment them (for example, classify potential non-productive transcripts according to whether they are seen more prominently in the RNA quality-control mutants).

***Q) For the reasons above, we reiterate that the source of transcripts is not important in the reference transcriptome. Whether transcripts such as NMD-sensitive transcripts come from the RNA processing mutant sample, wild-type samples or plants exposed to stresses is immaterial. In addition, the normal method to identify NMD substrates is to compare the abundance of transcripts in a mutant to wild-type and identify those transcripts with significantly increased abundance as NMD-sensitive. We only have one biological repeat of the mixed mutant sample such that quantitative analysis is not possible with any degree of confidence. Here, the mutants were used to increase the discovery of NMD or processed transcripts (which can be of low abundance depending on the gene/transcript) so that such transcripts can be readily identified when analysing replicated RNA-seq experimental data. Importantly, the program that characterises the transcripts (TranSuite; Entizne et al bioRxiv) incorporates transcript features from a number of Arabidopsis NMD publications including our own (Kalyna et al., 2012) and papers from the Silhavy lab AND generates accurate translations allowing identification of PTCs. This highlights the number of mis-annotated transcripts in TAIR10 and Araport11!***

2. While some attention is paid to possible NMD substrates, nonstop RNAs are not mentioned or identified. These would be apparent as transcripts with TES within protein-coding regions. This is an important subset of non-productive transcripts and should be identified and annotated in AtRTD3.

***R) Non-stop mRNAs do not contain a stop codon. They can be generated by premature polyadenylation, endonucleolytic cleavage of an mRNA in the coding region such as by miRNAs, or through point mutations which cause loss of the normal stop codon. The non-stop decay pathway has only recently been shown to function in plants and is not well studied.***

***The reasons that AtRTD3 is not a good platform for identifying non-stop mRNAs are 1) the main objective of the RTD is to allow accurate quantification of genes and transcripts in RNA-seq data; 2) we (and others) have shown that transcript fragments skew transcript quantification; 3) our short read quality control filters remove transcript fragments and our method to distinguish TSS and TES from degradation products also removes many transcript fragments; and 4) non-stop mRNAs are likely to be “seen” as transcript fragments and be removed.***

***Nevertheless, the data from the mutant sample is available for those with specific expertise in non-stop decay to help define NS mRNAs. Also, were a research group(s) to identify non-stop RNAs, these can be easily added to the RTD.***

3. In the methods, it is stated that one sample was prepared using RNA from isolated nuclei. While I can appreciate that this sample would expand the database to include unspliced or partially-spliced transcripts, it is not clear how this will inform a database of transcripts to be used by the Arabidopsis community. Intronic transcript sequences arising from nuclear RNA may or may not be relevant to aspects of gene regulation (alternative splicing and the like), and it seems as if this will only bloat the complete database. Perhaps transcript isoforms present largely or exclusively in the nuclear RNA samples can be so annotated, and perhaps segregated into a separate database.

***S) The reasons for using nuclei to generate RNA-seq data are that intron retention (IR) events are prevalent in plants, transcripts containing IRs are retained in the nucleus and avoid NMD, a subset of IR events are exitrons and alter the CDS of genes dependent on whether they are spliced or unspliced, and there are a growing number of examples where intron retention/detention regulates expression. Transcripts with IRs that have a regulatory role tend to have higher abundance than intermediates of splicing (e.g. see exitron papers – https://genome.cshlp.org/content/25/7/995.full) and the nuclear sample was included to capture such transcripts. To distinguish potential regulatory IR-containing transcripts in different conditions, it is important to accurately quantify transcripts which requires the RTD to be as comprehensive as possible. As stated above, partially spliced transcripts are resolved in analysis of RNA-seq experiments where they effectively flat-line at the bottom of expression plots. Their presence in the RTD is unlikely to affect quantification of the main transcript isoforms.***

***It is noteworthy that the nuclear RNA sample contributed greatly to the non-coding RNAs and, particularly, for novel genes in AtIso and AtRTD3. The nuclear RNA sample contributed the lowest number of genes and transcripts of all the samples (4,422 genes and 6,370 transcripts). Of these, 1,515 genes and 3,574 transcripts were unique to the nuclear RNA sample (Table S8) and 1,319 transcripts were non-coding RNAs. Finally, 912 genes and 1,073 transcripts from the nuclear RNA sample corresponded to the novel genes identified in AtIso and AtRTD3. These genes/transcripts most likely derive from RNAs that do not leave the nucleus and are lncRNA genes.***

4. There have been other reports describing chimeric transcripts in Arabidopsis (Duc. et al., PLoS Genet. 2013 Oct;9(10):e1003867 and Pontier et al., Life Sci Alliance. 2019 May 29;2(3):e201900393 come to mind). These reports might used to validate some of the chimeric RNAs reported in AtRTD3.

***T) We have compared the chimeric transcripts from AtRTD3 to those of Duc et al. (2012) and Pontier et al (2019) and find that a small number of the chimeric transcripts are common (page 23). It is important to note that these studies detected chimeric RNAs in the fpa mutant (Duc et al., 2013) or in the nerd1 mutant which affect correct termination of transcription of some genes allowing read-through into the downstream gene. Such mutants are not included in our analysis.***

5. TES's were validated using signals/motifs derived from characterizations of mRNA polyadenylation in mammals (pp. 16-17, Fig. 3E and F, noted in Table S6). Since plant 3' processing signals are somewhat different, it would be better to validate using different motifs or approaches.

***U) These 3’ processing motifs have been observed in multiple species, including plants and were used to validate TES in cotton long read data (Wang et al., 2019 Nature Communications 10:4714 https://doi.org/10.1038/s41467-019-12575-x). We think these are reasonable methods to validate plant TES data because, despite greater variability in 3’ processing signals in plants, there is conservation of some cis-elements and orthologues of cleavage and polyadenylation factors. For example, the polyadenylation signal, AAUAAA, was the most commonly occurring motif around preferred cleavage sites in Arabidopsis although many other variants of this sequence were also found (Sherstnev et al., 2012 Nat Struct Mol Biol. 19:845-52). Similarly, orthologues of eukaryotic cleavage/polyadenlation factors have been identified in plants (Hunt et al. BMC Genomics 2012, 13:641) including CFIm25 which is thought to bind a UGUAN motif. Although the binding site sequence has not been determined in plants, there is clear enrichment of this element upstream of the cleavage site of the TES determined by our methods.***

6. Several "new" transcripts are presented in the AtRDT3 database. Some additional study of these would seem to be warranted. For example, can possible function for predicted proteins be assigned? Is there evolutionary conservation of novel proteins or non-coding RNAs? Is there evidence, in other genome-scale or proteomic studies, of translation of these novel transcripts? Are there hints of tissue-specific or other regulated aspects of the expression of these transcripts?

***V) We have extracted all the novel genes identified in AtRTD3 and carried out functional annotation analysis using TRAPID 2.0 (http://bioinformatics.psb.ugent.be/trapid_02) (page22-23). Among the 1985 transcripts, best similarity search using DIAMOND identifies hits for 1320 transcripts from a range of plant species with 1131(85.68%) coming from Arabidopsis thaliana and 49 (3.71%) from Arabidopsis Lyrata. 897 gene families and 4 RNA families are identified among the hits. The structural and meta information for all the transcripts from the novel genes are shown in Supp Table S12. Amore complete functional analysis of the new transcripts is outside the scope of this paper and will be better addressed by groups with expertise in specific genes/genes families and the wherewithal to conduct functional analysis.***

***This is also not just an issue for the “new” transcripts here; there are still relatively few examples of functional differences being demonstrated for protein isoforms in plants largely due to the historical approach of working at the gene level instead of the transcript level. AtRTD3 provides detailed information on transcript isoforms and their coding capacity which will aid research groups to address functional diversity due to AS.***

**Second round of review**

**Reviewer 1**

I thank the reviewers for their considered response. Again, the technical aspects of the paper are very strong, and I only disagree on the interpretation. Biological systems are intrinsically noisy, and when I refer to “transcriptional noise”, I do mean real transcripts that are mis-spliced for instance, and not bioinformatic errors in assembly or quantification. Varabyou et al. showed that biological transcriptional noise (not just bioinformatic) negatively affects the quantification by Salmon and Kallisto. Therefore if one could list every single transcription intermediate, mis-spliced transcript, etc - real molecules that are found in cells (but very rare) - it could negatively impact isoform estimates by Salmon-Kallisto. This “comprehensive list” is what the authors aim for; the new saturation analysis (very useful, thank you) shows that we may need to increase the AtRTD3 transcript list multiple folds to get close to that goal. There must be a point where adding more and more aberrant (but real) transcripts isn’t helpful anymore. I think it’s an important point that should be discussed. The authors also hint at it in their reply to reviewer 2, on non-stop transcripts.  Aren’t these prematurely polyadenylated transcripts, or products of miRNA degradation, “real transcripts” that could fit the inclusion criteria, accumulate differently between conditions, and also have biological meaning? Shouldn’t we aim to catalogue them and quantify them? But the authors raise the issue that they bias the quantification. It seems to me to be the same issue highlighted by Varabyou et al.
It may very well be that the only way to do a good job at isoform quantification is to do long-read sequencing in the relevant system. As the authors point out, there could always be a special condition (biotic/abiotic stress, point mutation,...) that leads to the expression of a new isoform, and short reads won’t tell you.
Therefore I think there needs to be a deeper discussion on the utility of AtRTD3, how it’s still far from comprehensive, what transcriptional noise means and how it affects quantification. This was my point #3.

Let me clarify #4. I thought you could use AtRTD3 as the ground truth, quantify all the tpms for all the isoforms across a number of short RNA-seq experiments (e.g. the cold response time series), and check how may tpms were assigned to new isoforms compared to previous annotations. The actual ground truth isn’t known of course, but it’s still a good indication of how different the estimates would come out if one ran their short RNA-seq quantification against different annotations.

My minor comments on Figures have all been addressed, thank you.

**Reviewer 2**

For the most part, the authors have addressed my concerns in a satisfactory manner. I still do not agree with their decision to largely ignore non-stop RNAs. I believe it would be helpful to differentiate nonstop RNAs from other products of alternative TES that change the protein-coding capacity of the gene. Nonstop RNAs should not be confused with mRNAs that may yield appreciable quantities of alternative protein isoforms (which is what happens if one groups all coding region TES into a single set). It seems that it would be a very simple exercise to specifically note probable nonstop RNAs in one of the Supplementary Tables, much as has been done for possible NMD substrates and other non-productive RNAs. They likely are relevant for the regulation of expression of many important genes (one example - Parker et al., eLife. 2021 Apr 27;10:e65537) and their inclusion would expand the utility of the database in the community.

**Authors Response**

**Point-by-point responses to the reviewers’ comments:**

*Reviewer #2: For the most part, the authors have addressed my concerns in a satisfactory manner. I still do not agree with their decision to largely ignore non-stop RNAs. I believe it would be helpful to differentiate nonstop RNAs from other products of alternative TES that change the protein-coding capacity of the gene. Nonstop RNAs should not be confused with mRNAs that may yield appreciable quantities of alternative protein isoforms (which is what happens if one groups all coding region TES into a single set). It seems that it would be a very simple exercise to specifically note probable nonstop RNAs in one of the Supplementary Tables, much as has been done for possible NMD substrates and other non-productive RNAs. They likely are relevant for the regulation of expression of many important genes (one example - Parker et al., eLife. 2021 Apr 27;10:e65537) and their inclusion would expand the utility of the database in the community.*

We apologise for misunderstanding the reviewer's comment about providing a list of genes with non-stop mRNAs. Premature polyadenylation is an important mechanism in regulating gene expression as seen in the well characterised internal polyadenylation sites in FCA and FPA. We considered premature polyadenylation as alternative polyadenylation and mentioned in the text that our methods for detecting TES detected known internal polyadenylation sites in FCA and FPA and other genes rather than considering non-stop RNAs. This was because the plant literature has referred to premature polyadenylation or transcription termination for many years and only very recently has non-stop RNAs been mentioned in this regard (Szádeczky-Kardoss et al., 2018 and Parker et al 2021). We therefore thought the reviewer was referring to transcripts with endonucleolytic cleavage or with mutations in the authentic stop codon. We felt that the former would be rapidly degraded or lost in our quality control measures to remove fragments and that the latter would be very rare and difficult to distinguish in a single genetic background.

Non-stop RNAs are one outcome of premature polyadenylation and these transcripts are targeted for degradation by the non-stop decay pathway. We have now identified non-stop RNAs in AtRTD3. The challenge was not trivial but we have now generated a list of protein-coding genes with non-stop RNA transcripts (Additional File 1: Table S15A and B). The main class of genes are disease resistance genes.

We have made changes to the text to reflect these results and have added a table - Additional File 1: Table S15A and B - as the reviewer has requested. The method has been added to the Methods section.

*Reviewer's Responses to Questions*

*<b>Please provide your comments for the author(s) on the revised manuscript.</b></br>*

*Reviewer #1: I thank the reviewers for their considered response. Again, the technical aspects of the paper are very strong, and I only disagree on the interpretation. Biological systems are intrinsically noisy, and when I refer to “transcriptional noise”, I do mean real transcripts that are mis-spliced for instance, and not bioinformatic errors in assembly or quantification. Varabyou et al. showed that biological transcriptional noise (not just bioinformatic) negatively affects the quantification by Salmon and Kallisto. Therefore if one could list every single transcription intermediate, mis-spliced transcript, etc - real molecules that are found in cells (but very rare) - it could negatively impact isoform estimates by Salmon-Kallisto. This “comprehensive list” is what the authors aim for; the new saturation analysis (very useful, thank you) shows that we may need to increase the AtRTD3 transcript list multiple folds to get close to that goal. There must be a point where adding more and more aberrant (but real) transcripts isn’t helpful anymore. I think it’s an important point that should be discussed.*

*The authors also hint at it in their reply to reviewer 2, on non-stop transcripts.  Aren’t these prematurely polyadenylated transcripts, or products of miRNA degradation, “real transcripts” that could fit the inclusion criteria, accumulate differently between conditions, and also have biological meaning? Shouldn’t we aim to catalogue them and quantify them? But the authors raise the issue that they bias the quantification. It seems to me to be the same issue highlighted by Varabyou et al.*

*It may very well be that the only way to do a good job at isoform quantification is to do long-read sequencing in the relevant system. As the authors point out, there could always be a special condition (biotic/abiotic stress, point mutation,...) that leads to the expression of a new isoform, and short reads won’t tell you.*

*Therefore I think there needs to be a deeper discussion on the utility of AtRTD3, how it’s still far from comprehensive, what transcriptional noise means and how it affects quantification. This was my point #3.*

*Let me clarify #4. I thought you could use AtRTD3 as the ground truth, quantify all the tpms for all the isoforms across a number of short RNA-seq experiments (e.g. the cold response time series), and check how may tpms were assigned to new isoforms compared to previous annotations. The actual ground truth isn’t known of course, but it’s still a good indication of how different the estimates would come out if one ran their short RNA-seq quantification against different annotations.*

*My minor comments on Figures have all been addressed, thank you.*

The reviewer was concerned about the impact of “transcriptional noise” on the accuracy of transcript quantification by Salmon and kallisto and asked for a more detailed discussion of the utility of AtRTD3.

In particular, he/she was concerned that including transcripts that were real but likely noise would negatively affect the accuracy of quantification. The Varabyou et al 2021 paper looked at the impact of “splicing noise”, “intronic noise” and “intergenic noise” on quantification accuracy and concluded that >99% of the effects observed were due to “splicing noise” and that “intronic noise” and “intergenic noise” had little effect. The reviewer suggests that including potentially “aberrant” but “real” isoforms in our goal of making a very comprehensive transcript reference, will ultimately have a negative impact on transcript quantification as observed in the Varabyou paper.

There are a number of major reasons why this is unlikely to be the case with AtRTD3/Arabidopsis (note: we have analysed human RNA-seq data and have reasonable familiarity with human data).

1) Varabyou et al used human data while AtRTD3 is based on the Col-0 ecotype of Arabidopsis;
2) The human GTEx RNA-seq libraries used in Varabyou et al are from 54 tissues from nearly 1,000 individuals and GTEx data has collapsed isoforms for each gene. In contrast, AtRTD3 is constructed from different samples of the same genetic background (Col-0 ecotype) using the Col-0 genomic sequence;
3) The splicing data from the GTEx libraries will include splicing noise that derives from variation among different individuals with SNPs/indels affecting splicing of introns (cryptic splice sites, splice site mutations etc) in a wide range of genes. This is not the case for Arabidopsis Col-0 where all samples are from a single genetic background;
4) Intron size is massively different in human and Arabidopsis and is important when considering the impact of transcripts with intron retentions. In Arabidopsis the mean and median lengths of introns are 168 and 100 bp (Arabidopsis Genome Initiative. Nature. 2000;408:796–815) while the mean and median in human are 6938 bp and 1747 bp (Piovesan et al. BMC Res Notes 2019). The consequence is that intron retentions in Arabidopsis are captured within full-length transcripts while large intron retention events (tens of thousands of nt) in the human transcriptome are often represented by transcript fragments where the sequence runs into intron sequence and contains partial intron sequence;
5) The latter occur because in the human transcriptome most genes have full-length transcripts but some novel splicing features (e.g. alternative splice sites; cryptic splice sites; intron retention) come from annotation of ESTs and so are represented by transcript fragments. In contrast, in AtRTD3, we have removed transcript fragments because we recognised the negative impact of such fragments on quantification by Salmon and kallisto (Zhang et al 2017 – NAR).
6) “Transcriptional noise” and its cause are not well defined in the Varabyou paper. They defined “splicing noise” as “erroneous transcripts caused by the use of the wrong splice site”!!! This is in comparison to the “known transcripts” and the most commonly used splice sites. Thus “splicing noise” refers to 1) some novel splice junctions and 2) variation in splice site usage among the many different individuals (SNPs etc). Some of this variation is represented by transcript fragments which skew quantification. In AtRTD3, we do not have this type of genetic variation, transcript fragments have been removed and transcripts are full-length.

In summary, in AtRTD3, transcript fragments have been removed, splicing variants in isoforms including intron retentions are represented by full-length transcripts, and the RNA-seq libraries are from the same genetic background. Therefore, the issue of “splicing noise” is unlikely to affect quantification of transcripts.

We have added a more detailed discussion to the Discussion section.

In regard to #4, it is very difficult to compare directly transcripts in the different annotations and therefore to address what degree of expression is associated with new isoforms (due to some isoforms from short reads assembly has the issue of combining parts of real transcript making artificial hybrids). We showed improved resolution of expression contribution between AtRTD2 and AtRTD3 for specific genes in Figures S15-17 reflecting improved transcript definition (S15 and S16) and missing isoforms in AtRTD2 (S17). We have recently analysed a splicing factor mutant versus Col-0 wild-type control using AtRTD2 and AtRTD3 to quantify transcripts. With AtRTD3 we observed a ca. 20% increase in differentially expressed genes and four-fold increase in differentially alternatively spliced genes which reflects the impact of the novel isoforms.
